# Distress and Its Determinants in 820 Consecutive Melanoma Patients

**DOI:** 10.1002/cam4.70820

**Published:** 2025-03-24

**Authors:** Markus Reitmajer, Petra Riedel, Claus Garbe, Norbert Schäffeler, Thomas K. Eigentler, Andrea Forschner

**Affiliations:** ^1^ Department of Dermatology University Hospital Tuebingen Tuebingen Germany; ^2^ Department of Psychosomatic Medicine and Psychotherapy University Hospital Tübingen Tübingen Germany; ^3^ Department of Dermatology Charité ‐ Universitätsmedizin Berlin, Corporate Member of Freie Universität Berlin and Humboldt‐Universität zu Berlin Berlin Germany

**Keywords:** Distress Thermometer, melanoma, NCCN Problem List, psycho‐oncological burden, psycho‐oncological support, screening tool

## Abstract

**Background:**

Psycho‐oncological burden not only affects patients' mental health but can also decrease treatment compliance and impair outcomes. The Distress Thermometer (DT) is a widely used screening tool in real‐world medical care for identifying and monitoring psychological distress. Patients with melanoma presenting in oncologic outpatient departments comprise a wide range of characteristics. Although young adults may face challenges related to pivotal life stages, such as career responsibilities or parenting, older adults often contend with mobility issues, preexisting comorbidities, or age‐related physical limitations.

**Methods:**

We conducted a retrospective evaluation of DT data from 820 patients with melanoma treated at our outpatient department between July and September 2016. These patients underwent routine DT screening and completed the associated National Comprehensive Cancer Network (NCCN) Problem List. The study aimed to identify factors influencing DT values above the threshold (≥ 5), further characterizing the patients' complaints according to the NCCN Problem List.

**Results:**

A total of 820 patients with melanoma underwent psycho‐oncological screening. More than 40% had DT values above the threshold. Significant factors associated with DT values over the threshold included female gender, younger age, and advanced melanoma stages III–IV. Analysis of the NCCN Problem List revealed complaints such as fear, sleep issues, tingling in hands and feet, feeling swollen, problems at work or school, concerns regarding God, and loss of faith.

**Conclusion:**

The results indicate a high need for psycho‐oncological support for patients with melanoma. Particular attention should be given to patients with the identified factors that are associated with exceeding the DT threshold.

## Graphical abstract

Psycho‐oncological distress is common in melanoma patients, with over 40% showing elevated distress levels (DT ≥ 5), particularly among women, younger individuals, and those with advanced disease stages (III–IV). Key complaints identified through the NCCN Problem List include fear, sleep disturbances, physical symptoms, and existential concerns. These findings emphasize the importance of targeted psycho‐oncological support, especially for high‐risk patient groups.

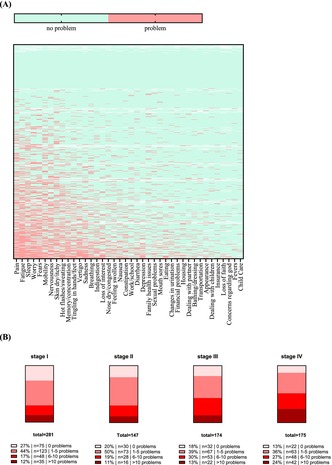

## 1 Background

The total number of patients with cancer is rapidly increasing, not only due to rising incidence rates but also because more patients are surviving long‐term [1]. However, an estimated 50% of patients with cancer experience emotional distress, reduced quality of life, and significant psychosocial challenges [2, 3, 4]. Approximately one‐third of patients with cancer are affected by depression, anxiety, or adjustment disorders [5]. This psychological burden can decrease treatment compliance and may negatively impact treatment outcomes [6, 7]. However, the psycho‐oncological status may change over time. It has been shown that although more than 50% of patients were above the threshold on the DT at the beginning of immune checkpoint inhibitor (ICI) treatment, indicating a need for psycho‐oncological support, this rate dropped to around 40% during the course of treatment [8]. Therefore, a careful assessment of psycho‐oncological distress seems reasonable. Psycho‐oncological support should be offered to patients with increased distress, with evidence of effective improvement in quality of life [9, 10, 11]. The Distress Thermometer (DT) is a widely used screening tool for identifying psychological distress across various types of cancer and for monitoring psycho‐oncological distress in routine daily care and throughout treatment [12, 13]. The DT has been validated by numerous studies in different types of cancer. In a large meta‐analysis of 42 studies, the pooled sensitivity of the DT was 81% (95% CI: 0.79–0.82) and the pooled specificity was 72% (95% CI: 0.71–0.72), making it a reliable screening tool [14].

However, not all patients with cancer face the same circumstances. Different types of cancer can vary significantly in terms of patient age, treatment approaches, and prognosis. Melanoma is one of the most frequently diagnosed cancers in individuals aged 15–29, although it predominantly affects older individuals [15, 16, 17, 18]. Adolescent and young adult patients are often in a phase of life where career responsibilities and other social roles, such as parenting, are important [15, 18]. This challenging situation can rise up notable psychological and social challenges. To date, known predictors for distress in melanoma are practical problems at work, family‐related problems (dealing with the partner) and physical problems (pain, appearance, nausea) [19]. The aim of this study was to explore in a cohort of 820 patients with melanoma further factors which may have led to DT values over threshold and to characterize patients' complaints and burden according to the NCCN Problem List.

## 2 Methods

### 2.1 Study Design

We conducted a retrospective evaluation of DT data from patients with melanoma treated at our outpatient department between July and September 2016. All of these patients underwent routine screening using the DT and were additionally asked to complete the associated National Comprehensive Cancer Network (NCCN) Problem List. DT values range from 0 (no distress) to 10 (extreme distress), with scores of ≥ 5 considered above the threshold, indicating the need for psycho‐oncological support [20, 21, 22]. The NCCN Problem List addresses a wide spectrum of practical, family, emotional, physical, and spiritual issues and is often used in conjunction with the DT [12, 20, 22]. The psycho‐oncological screening was performed prior to the patients' consultations with the physician.

### 2.2 Eligibility Criteria and Ethical Approval

The eligibility criteria included: patients with melanoma with a histologically confirmed diagnosis of melanoma, consent to participate, age of 18 years or older, and the ability to read and understand questionnaires. Ethical approval was obtained by the University of Tuebingen (reference number 009/2017BO2), and the study was conducted according to the Declaration of Helsinki.

### 2.3 Statistical Analysis

The data were assessed by IBM SPSS Statistics (version 28.0.0.0.). Statistical analysis was performed with IBM SPSS Statistics (version 28.0.0.0). Incomplete questionnaires were included with the completed answers. Patient data were extracted from electronic medical records. Descriptive analysis was performed to characterize the patients characteristics regarding gender, median time since diagnosis in months, median age in years, melanoma subtype (cutaneous, occult, acral lentiginous, uveal and mucosal), American Joint Committee on Cancer (AJCC) stage at the time of the survey, AJCC stage at the time of initial diagnosis, and potentially applied systemic therapies (ICI, targeted therapy (TT), chemotherapy), as well as to assess the values of the DT and the number of reported complaints on the NCCN Problem List. Binary logistic regression analysis was performed to identify potential risk factors in patient‐specific data and reported NCCN Problem List concerns indicating the need for psycho‐oncological support. Graphs were generated using GraphPad PRISM 9.5.0 (Dotmatics, Boston, USA).

## 3 Results

### 3.1 Patient Characteristics

A total of 820 patients with melanoma were included. The cohort consisted of 406 men (49.5%) and 414 women (50.5%), with a median age of 63 years (ranging from 21 to 94 years) and a median time since melanoma diagnosis of 38 months (ranging from the month of diagnosis to 38 years after initial diagnosis). Among them, 359 patients (44%) had advanced melanoma (stage III: *n* = 182; stage IV: *n* = 177), whereas the remaining 56.2% had stage I (*n* = 308) or stage II (*n* = 153) melanoma. Systemic treatment for metastases was applied to 120 patients (14.6%), of whom 90 (75%) received ICI, 27 patients (22.5%) received TT, and three patients (0.03%) received chemotherapy. Cutaneous melanoma was the most frequent type of melanoma in the cohort, with 651 patients (79.4%), followed by acral lentiginous melanoma with 39 patients (4.8%), occult melanoma with 35 patients (4.3%), uveal melanoma with 18 patients (2.2%), and mucosal melanoma with 14 patients (1.7%) (Table 1).

**TABLE 1:** Patients' characteristics.

Patient's characteristics	*N* (%)
Total	820 (100)
Sex	
Female	414 (50.5)
Male	406 (49.5)
Median age in years [range]	63 [21–94]
Median time since diagnosis in months [range]	38 [0–457]
AJCC stage at the time of survey	
AJCC stage I	308 (37.6)
AJCC stage II	153 (18.7)
AJCC stage III	182 (22.2)
AJCC stage IV	177 (21.6)
AJCC stage at the time of initial diagnosis	
AJCC stage I	363 (44.3)
AJCC stage II	232 (28.3)
AJCC stage III	172 (21.0)
AJCC stage IV	18 (2.2)
No data available	35 (4,3)
Patients under systemic treatment	120 (14.6)
Immune checkpoint inhibitors	90 (11)
Targeted therapy	27 (3.3)
Chemotherapy	3 (0.4)
Melanoma type	
Cutaneous	651 [79.4]
Occult	35 [4.3]
Acral lentiginous	39 [4.8]
Uveal	18 [2.2]
Mucosal	14 [1.7]
Not histologically differentiated	63 [7.7]
DT values	
DT values ≤ 4 (normal)	482 (58.8)
DT values ≥ 5 (above the threshold)	338 (41.2)

Abbreviations: AJCC, American Joint Committee on Cancer; DT, Distress Thermometer; *N*, number of patients.

### 3.2 More Than 40% of the Patients Had DT Values Over Threshold

**FIGURE 1:**
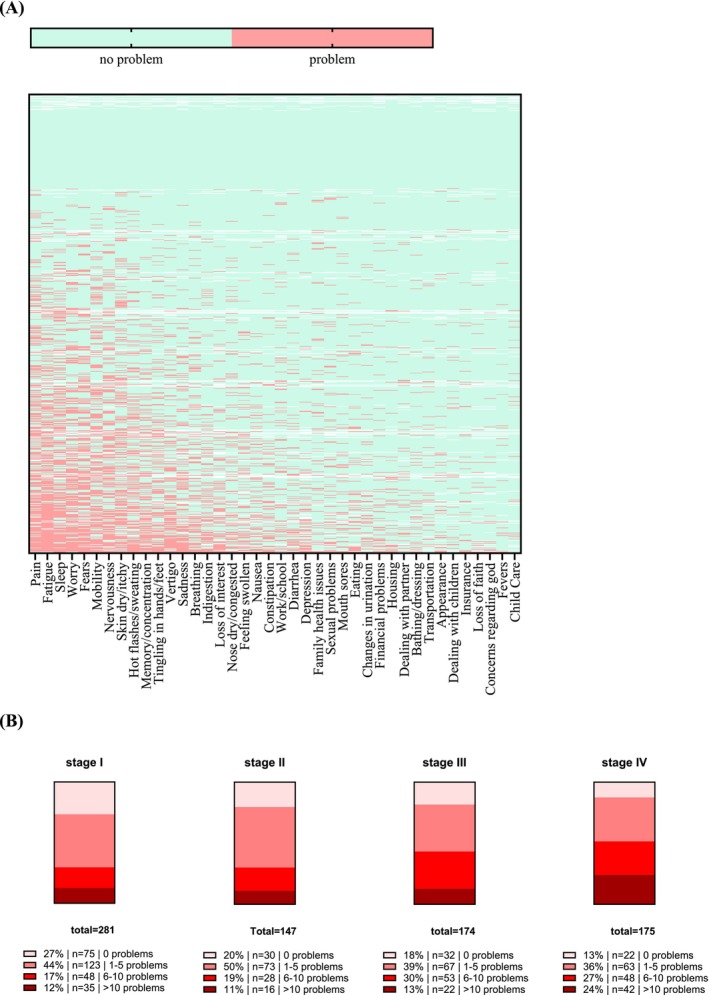
(A) Heat map provides a visual overview of all issues identified by all 777 patients with melanoma who completed the NCCN questionnaire. Horizontal axis: the most frequent problems are arranged along the left side of the heat map. Vertical axis: patients experiencing the most problems are located toward the bottom of the heat map. This leads to an accumulation of patients with multiple issues (bottom), creating an impression of which NCCN problems are common in this cohort (left side) and which are not (right side). (B) Patients were categorized into four groups on the basis of the NCCN Problem List: Group 1 reported 0 problems on the NCCN Problem List, Group 2 reported 1–5 problems, Group 3 reported 6–10 problems, and Group 4 reported > 10 problems.

Overall, 338 patients (41.2%) had DT values over threshold, whereas 482 patients had DT values of ≤ 4. Physical concerns were the most frequently reported problem group on the NCCN Problem List (Table 2). More than 30% of patients reported pain (36.2%), fatigue (35.7%), sleep problems (31.9%) or worry (30.3). Nearly 30% mentioned issues such as fears (29.8%), mobility problems (29.1%), nervousness (28.1%), and dry/itchy skin (27.9%) (Table 2). A comprehensive overview of all results from the NCCN Problem List is provided in Figure 1 and supplementary Table S1. The 10 most frequently mentioned concerns are shown in Table 2.

**TABLE 2:** Ten most frequently mentioned concerns.

Problem	Category of concern	Yes | No	*N*	%	DT ≤ 4 | *N* (%)	DT ≥ 5 | *N* (%)
Pain	Physical	No	470	63.8	312 (66.4)	158 (33.6)
		Yes	267	**36.2**	111 (41.6)	156 (58.4)
Fatigue	Physical	No	468	64.3	325 (69.4)	143 (30.6)
		Yes	260	**35.7**	93 (35.8)	167 (64.2)
Sleep	Physical	No	495	68.1	336 (67.9)	159 (32.1)
		Yes	232	**31.9**	83 (35.8)	149 (64.2)
Worry	Emotional	No	510	69.7	357 (70)	153 (30)
		Yes	222	**30.3**	72 (32.4)	150 (67.6)
Fears	Emotional	No	511	70.2	361 (70.6)	150 (29.4)
		Yes	217	**29.8**	66 (30.4)	151 (69.6)
Mobility	Physical	No	519	70.9	336 (64.7)	183 (35.3)
		Yes	213	**29.1**	93 (43.7)	120 (56.3)
Nervousness	Emotional	No	525	71.9	358 (68.2)	167 (31.8)
		Yes	205	**28.1**	70 (34.1)	135 (65.9)
Skin dry/itchy	Physical	No	531	72.1	327 (61.6)	204 (38.4)
		Yes	205	**27.9**	101 (49.3)	104 (50.7)
Hot flashes/sweating episodes	Physical	No	560	76.5	355 (63.4)	205 (36.6)
		Yes	510	**23.5**	357 (70)	153 (30)
Memory/concentration	Physical	No	587	80.2	371 (63.2)	216 (36.8)
		Yes	145	**19.8**	55 (37.9)	90 (62.1)

Abbreviations: %, percentage of cohort; DT, Distress Thermometer; *N*, number of patients. Bold values indicate the percentage of patients who reported experiencing the respective problem. The color shading represents the categories of concern: blue for physical problems, red for emotional problems.

### 3.3 Factors Significantly Associated With DT Values Over Threshold

Binary logistic regression identified both patient characteristics and specific concerns from the NCCN Problem List to be significantly associated with DT values over threshold (DT ≥ 5), thus indicating a need for psycho‐oncological support. Among all assessed patient characteristics, the four significant factors for distress values over threshold included female gender (OR = 1.420, *p* = 0.016), increasing age (OR = 0.985, *p* = 0.002), advanced melanoma stage III (OR = 1.567, *p* = 0.023) and melanoma stage IV (OR = 2.602, *p* = 0.001), whereas factors such as longer time since diagnosis (OR = 0.998, *p* = 0.110), melanoma subtype (OR = 0.967, *p* = 0.602), and systemic treatment (OR = 0.695, *p* = 0.248) were not significantly associated with higher DT values (Table 3).

**TABLE 3:** Summary of logistic regression on DT values ≥ 5.

	*B*	SE	Wald	*p*	OR	95% CI for OR	Cox & Snell	Nagelkerke
Patients' characteristics								
Gender female vs. male	0.351	0.146	5.762	0.016*	1.420	1.066–1.890	*R* ^2^ = 0.36	*R* ^2^ = 0.49
AJCC stage III vs. AJCC stage I	0.449	0.197	5.196	0.023*	1.567	1.065–2.305		
AJCC stage IV vs. AJCC stage I	0.956	0.297	10.362	0.001*	2.602	1.454–4.658		
Increasing age of patients	−0.015	0.005	9.486	0.002*	0.985	0.975–0.994		
Longer time since first diagnosis	−0.002	0.001	2.558	0.110	0.998	0.995–1.000		
Systemic treatment yes vs. no	−0.364	0.315	1.334	0.248	0.695	0.374–1.289		
Cutaneous melanoma type vs. another melanoma type	−0.032	0.062	0.273	0.602	0.968	0.858–1.093		
NCCN Problem List concerns								
Emotional problems							*R* ^2^ = 0.29	*R* ^2^ = 0.40
Worry	0.507	0.329	2.378	0.123	1.660	0.872–3.160		
Fears	0.507	0.329	2.378	0.004*	2.677	1.371–5.227		
Sadness	0.985	0.341	8.317	0.093	1.886	0.899–3.957		
Depression	0.634	0.378	2.816	0.950	1.039	0.311–3.475		
Nervousness	0.038	0.616	0.004	0.350	1.337	0.727–2.457		
Loss of interest in usual activities	0.290	0.311	0.872	0.056	2.452	0.978–6.148		
Spiritual problems								
Concerns regarding God	0.897	0.469	3.655	0.030*	0.030	0.001–0.710		
Loss of faith	−3.512	1.617	4.718	0.042*	12.717	1.093–147.893		
Physical problems								
Pain	2.543	1.252	4.126	0.219	1.440	0.805–2.578		
Nausea	0.365	0.297	1.510	0.148	2.135	0.763–5.971		
Fatigue	0.758	0.525	2.088	0.083	1.703	0.933–3.108		
Sleep	0.533	0.307	3.011	0.006*	2.207	1.262–3.861		
Mobility	0.792	0.285	7.699	0.672	1.145	0.612–2.141		
Bathing/dressing	0.135	0.319	0.180	0.772	0.783	0.150–4.091		
Appearance	−0.245	0.844	0.084	0.337	2.405	0.401–14.427		
Breathing	0.878	0.914	0.922	0.852	0.931	0.441–1.967		
Mouth sores	−0.071	0.381	0.035	0.158	0.455	0.152–1.359		
Eating	−0.788	0.559	1.992	0.947	1.060	0.191–5.889		
Indigestion	0.058	0.875	0.004	0.861	1.094	0.401–2.982		
Constipation	0.089	0.512	0.031	0.273	0.533	0.173–1.643		
Diarrhea	−0.630	0.575	1.200	0.322	0.585	0.203–1.689		
Changes in urination	−0.536	0.541	0.981	0.562	1.375	0.469–4.035		
Fevers	0.319	0.549	0.337	0.172	4.051	0.545–30.129		
Skin dry/itchy	1.399	1.024	1.867	0.211	0.679	0.371–1.245		
Nose dry/congested	−0.387	0.309	1.566	0.236	0.574	0.230–1.436		
Tingling in hands/feet	−0.554	0.467	1.407	0.011*	2.383	1.219–4.660		
Feeling swollen	0.868	0.342	6.445	0.034*	2.713	1.078–6.831		
Hot flashes/sweating episodes	0.998	0.471	4.490	0.234	0.674	0.352–1.291		
Vertigo	−0.394	0.331	1.416	0.565	0.805	0.385–1.686		
Memory/concentration	−0.217	0.377	0.330	0.696	0.862	0.409–1.817		
Sexual problems	−0.149	0.381	0.153	0.550	0.761	0.312–1.859		
Practical problems								
Housing	−0.272	0.455	0.358	0.684	0.702	0.128–3.850		
Insurance	−0.353	0.868	0.166	0.269	2.538	0.487–13.227		
Work/school	0.931	0.842	1.222	0.003*	3.958	1.580–9.917		
Transportation	1.376	0.469	8.617	0.244	2.132	0.596–7.625		
Financial problems	0.757	0.650	1.357	0.979	0.983	0.270–3.575		
Child care	−0.017	0.659	0.001	0.823	0.820	0.144–4.655		
Family health issues	−0.198	0.886	0.050	0.771	1.138	0.477–2.719		
Dealing with partner	0.130	0.444	0.085	0.964	1.032	0.264–4.034		
Dealing with children	0.032	0.696	0.002	0.312	2.042	0.512–8.148		

Abbreviations: *B*, regression coefficient; CI, 95% confidence Interval for the OR; OR, odds ratio; SE, standard error; Wald, Wald statistic.

* Significant and independent variables.

Binary logistic regression of the NCCN Problem List (Table 3) revealed seven additional concerns that were associated with DT values over thresholds. Among emotional concerns, fears (OR = 2.677, *p* = 0.004) emerged as a significant factor. In the physical category, significant issues included sleep problems (OR = 2.207, *p* = 0.006), tingling in hands/feet (OR = 2.383, *p* = 0.011), and feeling swollen (OR = 0.034, *p* = 0.034). For practical concerns, problems at work/school (OR = 3.958, *p* = 0.003) were significant. In the spiritual category, concerns regarding God (OR = 0.030, *p* = 0.030) and loss of faith (OR = 12.717, *p* = 0.042) were identified as significant indicators.

## 4 Discussion

Psycho‐oncological care has become an integral part of oncological care in most oncology outpatient departments of Germany. However, identifying exactly these patients who are in need of psycho‐oncological support might be challenging in daily clinical practice. Physicians' responsibilities usually focus on medical care and oncological issues and only rarely assess economic or social needs [23, 24]. In a study involving 620 patients with cancer in China, the average consultation duration between patients and physicians was < 5 min [25]. A similar study was conducted in the USA with evaluation of 2.470 physician–patient contacts with the result of a mean duration of patients' visits of < 23 min [26]. Both studies associated performance‐based payment mechanisms with a decrease in the amount of time physicians can spend with their patients [25, 26]. Many countries face an aging population, increasing prevalence of chronic illness, and increasing healthcare costs, assuming that a rapid and efficient identification of patients who require psycho‐oncological support becomes increasingly important [27, 28, 29]. In addition, new therapies (ICI and TT) may be associated with serious, potentially irreversible or chronic adverse events that could lead to increased psychosocial distress, making early identification important [30, 31]. Early identification of psychosocial distress also appears to be crucial, as there is evidence that chronic distress may adversely affect response rates to ICI. I [32, 33, 34]. The DT and the accompanying NCCN Problem List are screening tools that can quickly identify patients who need psycho‐oncology support [12, 22]. This could provide the opportunity to act and include these patients in specialized, evidence‐based programs for patients with cancer with distress, such as psychoeducational interventions and cognitive‐behavioral therapy [35, 36]. In the present study, we explored in a cohort of 820 patients with melanoma potential factors associated with DT values over threshold, and we aimed to characterize patients' complaints and burdens according to the NCCN Problem List.

Patients with melanoma in oncologic outpatient departments tend to be more diverse than patients with other cancer entities. Unlike prostate, cervical, or ovarian cancer, melanoma affects both women and men, although it is more frequent in men [37]. Most recently, an increasing incidence trend was found in older patients and males [38]. Second, survival rates vary widely depending on the stage of the disease. While in stage I melanoma, the 5‐year survival rate is approximately 99%, this rate drops to approximately 30% in stage IV [39, 40, 41]. Third, melanoma is not uncommon in younger patients, as it ranks among the most frequent cancers in young adults, although it predominantly affects older individuals [16, 18]. With the approval of new therapies for stage IIB‐IV melanoma, such as TT and ICI in both the adjuvant and palliative settings, the heterogeneity among patients with melanoma has increased even further [42, 43, 44, 45]. This cohort reflects the challenge of heterogeneity in a dermatologic oncology outpatient setting, with patients ranging in age from 21 to 94 and without gender predominance.

To date, known predictors for distress in melanoma include practical problems at work, family‐related problems (dealing with the partner) and physical problems such as pain, appearance concerns, and nausea [19]. Although pain was among the most frequently reported issues in our cohort, it was not significantly associated with distress values over the threshold in our binary regression model. However, we were able to confirm that problems at work or school were significantly associated with distress in our cohort, as was younger age. Regarding younger age, it is known that adolescents and young adults (15–39 years) with cancer often experience a lack of age‐adapted psychosocial support and health services, which might improve both quality of life and clinical outcomes [17]. Given that melanoma is not uncommon in this age group, and that this factor has been identified as significantly associated with exceeding the distress threshold, clinicians should pay particular attention to young patients during consultations. However, the statistical impact is small in our cohort, with an OR of 0.985, close to one, compared with a previously published cohort [19].

Additionally, we identified new factors that were correlated with DT values over the threshold in this study. Advanced stages III–IV compared with stage I were significant factors for DT values over the threshold. Both possible concerns in the spiritual category—concerns regarding God and loss of faith—were also significant factors. Interestingly, while a loss of faith is associated with exceeding the DT threshold, the OR for concerns regarding God indicates a decreased likelihood of exceeding this threshold. Although both factors are within the same concern group, they exhibit opposite effects. However, it is worth noting that the number of patients reporting spiritual issues is relatively low, with only 18 patients (reporting concerns regarding God) and 19 patients (reporting loss of faith).

A recent study on survivors of localized melanoma identified high rates of fear of cancer recurrence [46]. The explicit fear of recurrence was not addressed in our study; however, we assessed fear in general as a frequently reported issue (affecting approximately 30% of the cohort) and also as a significant factor for DT over threshold. Worrying and nervousness, similarly reported by approximately 30% of the cohort, were not associated with DT values over threshold. In total, more than 40% of patients in our cohort had DT values above the threshold, aligning with estimates that about 50% of patients with cancer experience emotional distress, reduced quality of life, and significant psychosocial challenges [2, 3, 4]. However, it is important to note that the DT is not the only tool available to assess psychological distress in patients with skin cancer. Other screening instruments, such as the Hospital Anxiety and Depression Scale (HADS) or the Hornheide Questionnaire (HQ), have also been shown to be effective in identifying the need for psychosocial support in patients with melanoma [47, 48].

### 4.1 Clinical Implication

Psycho‐oncological care should be an integral part of every oncological outpatient department. However, the ability of physicians to identify patients with the need for psycho‐oncological support is limited due to a lack of time and experience. In the present cohort of 820 patients with melanoma, a total of 11 factors were significantly associated with DT values over threshold. Although factors such as younger age and impairment in work or school had already been reported, additional factors such as female gender, fears, spiritual aspects, and sleep issues were newly identified. We aim with this study to raise awareness for patients by checking the identified factors when counseling patients with melanoma in daily routine. If conducting a psycho‐oncological assessment for every patient is not feasible in practice, particular attention should be given to patients belonging to the identified risk groups. We identified female gender, younger age, advanced melanoma stages III–IV, and concerns listed on the NCCN Problem List—such as fear, sleep issues, tingling in the hands and feet, feelings of swelling, problems at work or school, concerns about God, and loss of faith—as potential risk factors.

### 4.2 Limitations and Conclusion

It should be acknowledged that this study was conducted in Germany, where legally regulated social protection mechanisms, including health insurance, are established. Rates of practical and social concerns regarding insurance, housing, or financial problems may be higher in countries with less comprehensive social care systems. The influence of other comorbidities was not investigated in this cohort. Regarding the DT and the accompanying NCCN Problem List, it is important to note that they serve as self‐assessments, allowing patients to report their distress and problems without an objective evaluation by a third party. The timing of the assessment may therefore influence the DT, and for patients in a phase of denial, the results may not accurately reflect their objective or true situation.

Nevertheless, the study provides relevant real‐world data for the implementation and further development of psychosocial care within the heterogeneous group of 820 patients with melanoma. Further studies are needed to examine and characterize the identified issues more closely, such as fears, sleep issues, and recognized spiritual aspects. Additionally, research should focus more on the subgroup of adolescents and young adults to better understand the factors that may contribute to improving their quality of life and their work or school environment, as well as to adapt psychosocial care accordingly.

## Author Contributions

**Markus Reitmajer:** conceptualization (equal), data curation (equal), formal analysis (equal), investigation (equal), methodology (equal), software (equal), visualization (lead), writing – original draft (lead). **Petra Riedel:** writing – review and editing (equal). **Claus Garbe:** writing – review and editing (equal). **Norbert Schäffeler:** writing – review and editing (equal). **Thomas K. Eigentler:** writing – review and editing (equal). **Andrea Forschner:** conceptualization (equal), data curation (equal), formal analysis (equal), investigation (equal), methodology (equal), supervision (equal), validation (equal), writing – original draft (equal).

## Conflicts of Interest

M.R. received travel support from Almirall Hermal, Pierre Fabre, and Galderma, outside the submitted work. C.G. reports grants from Novartis, NeraCare, BMS, Roche, and Sanofi, and honoraria from MSD, Novartis, NeraCare, BMS, Philogen, Roche, and Sanofi outside the submitted work. T.K.E. received consulting fees from Bristol‐Myers Squibb, Novartis, Pierre Fabre, Regeneron, MSD, CureVac, Anaveon, and Sanofi, outside the submitted work. A.F. served as a consultant to Novartis, Merck Sharp & Dohme, Bristol‐Myers Squibb, Pierre Fabre, and Immunocore; received travel support from Novartis, BMS, and Pierre Fabre; received speaker fees from Novartis, Bristol‐Myers Squibb, and Merck Sharp & Dohme; and reports institutional research grants from Bristol‐Myers Squibb Stiftung Immunonkologie, outside the submitted work. The other authors declare that they have no conflicts of interest.

## Supporting information


Table S1.

## Data Availability

The datasets used and/or analyzed during the current survey are available from the corresponding author upon reasonable request.
