# Supplementary material for: Distress and Its Determinants in 820 Consecutive Melanoma Patients
Source: Cancer Med. 2025 Mar 24;14(6):e70820. doi: 10.1002/cam4.70820 (PMC11931323; doi:10.1002/cam4.70820)
Supplement: Supplementary file 1 — Table S1. [file CAM4-14-e70820-s001.docx]

# Supplements

## Supplementary table 1 Results of the NCCN-Problem List

For each subgroup, the 10 most frequent concerns were highlighted and color-coded by category. Following abbreviations are used: number of patients (N), percentage of cohort (%).

| **Problem** | **Category of concern** | **<45 years** | | **46-65 years** | | **>65 years** | | **whole cohort** | |
| --- | --- | --- | --- | --- | --- | --- | --- | --- | --- |
|  |  | **N** | **%** | **N** | **%** | **N** | **%** | **N** | **%** |
| **Housing** | **Practical** | 8/97 | 8.2 | 19/312 | 6.1 | 14/342 | 4.1 | 41/751 | 5.5 |
| **Insurance** |  | 4/97 | 4.1 | 12/311 | 3.9 | 12/334 | 3.6 | 28/742 | 3.8 |
| **Work/school** |  | 20/95 | 21.1 | 48/309 | 15.5 | 0/324 | 0.0 | 68/728 | 9.3 |
| **Transportation** |  | 2/96 | 2.1 | 12/305 | 3.9 | 20/323 | 6.2 | 34/724 | 4.7 |
| **Financial problems** |  | 10/95 | 10.5 | 30/306 | 9.8 | 5/334 | 1.5 | 45/735 | 6.1 |
| **Child care** |  | 9/98 | 9.2 | 3/302 | 1.0 | 5/323 | 1.5 | 17/723 | 2.4 |
| **Family health issues** |  | 3/94 | 3.2 | 41/304 | 13.5 | 19/334 | 5.7 | 63/732 | 8.6 |
| **Dealing with partner** |  | 10/96 | 10.4 | 18/303 | 5.9 | 12/333 | 3.6 | 40/732 | 5.5 |
| **Dealing with children** |  | 4/91 | 4.4 | 20/303 | 6.6 | 7/329 | 2.1 | 31/723 | 4.3 |
| **Worry** | **Emotional** | 42/94 | 44.7 | 114/302 | 37.7 | 66/336 | 19.6 | 222/732 | 30.3 |
| **Fears** |  | 39/93 | 41.9 | 99/301 | 32.9 | 79/334 | 23.7 | 217/728 | 29.8 |
| **Sadness** |  | 17/93 | 18.3 | 66/300 | 22.0 | 49/326 | 15.0 | 132/719 | 18.4 |
| **Depression** |  | 8/91 | 8.8 | 34/294 | 11.6 | 19/322 | 5.9 | 61/707 | 8.6 |
| **Nervousness** |  | 33/93 | 35.5 | 82/302 | 27.2 | 90/335 | 26.9 | 205/730 | 28.1 |
| **Loss of interst in usual acitvity** |  | 12/92 | 13.0 | 38/296 | 12.8 | 38/321 | 11.8 | 88/709 | 12.4 |
| **Concerns regarding God** | **Spiritual** | 2/95 | 2.1 | 9/285 | 3.2 | 7/325 | 2.2 | 18/705 | 2.6 |
| **Loss of faith** |  | 4/94 | 4.3 | 9/286 | 3.1 | 6/321 | 1.9 | 19/701 | 2.6 |
| **Pain** | **Physical** | 32/94 | 34.0 | 115/307 | 37.5 | 120/336 | 35.7 | 267/737 | 36.2 |
| **Nausea** |  | 9/94 | 9.6 | 35/308 | 11.4 | 28/331 | 8.5 | 72/733 | 9.8 |
| **Fatigue** |  | 37/92 | 40.2 | 124/302 | 41.1 | 99/334 | 29.6 | 260/728 | 35.7 |
| **Sleep** |  | 28/91 | 30.8 | 112/304 | 36.8 | 92/332 | 27.7 | 232/727 | 31.9 |
| **Mobility** |  | 14/93 | 15.1 | 86/307 | 28.0 | 113/332 | 34.0 | 213/732 | 29.1 |
| **Bathing/Dressing** |  | 2/94 | 2.1 | 13/306 | 4.2 | 23/330 | 7.0 | 38/730 | 5.2 |
| **Appearance** |  | 3/92 | 3.3 | 16/303 | 5.3 | 14/322 | 4.3 | 33/717 | 4.6 |
| **Breathing** |  | 4/95 | 4.2 | 46/306 | 15.0 | 63/327 | 19.3 | 113/728 | 15.5 |
| **Mouth sores** |  | 6/96 | 6.3 | 24/303 | 7.9 | 26/328 | 7.9 | 56/727 | 7.7 |
| **Eating** |  | 4/94 | 4.3 | 27/307 | 8.8 | 25/329 | 7.6 | 56/730 | 7.7 |
| **Indigestion** |  | 8/95 | 8.4 | 40/310 | 12.9 | 50/329 | 15.2 | 98/734 | 13.4 |
| **Constipation** |  | 1/94 | 1.1 | 25/308 | 8.1 | 43/327 | 13.1 | 69/729 | 9.5 |
| **Diarrhea** |  | 6/95 | 6.3 | 35/306 | 11.4 | 24/329 | 7.3 | 65/730 | 8.9 |
| **Changes in urination** |  | 1/95 | 1.1 | 15/308 | 4.9 | 36/328 | 11.0 | 52/731 | 7.1 |
| **Fevers** |  | 2/95 | 2.1 | 14/310 | 4.5 | 2/329 | 0.6 | 18/734 | 2.5 |
| **Skin dry/itchy** |  | 29/93 | 31.2 | 66/304 | 21.7 | 110/339 | 32.4 | 205/736 | 27.9 |
| **Nose dry/congested** |  | 10/94 | 10.6 | 38/303 | 12.5 | 37/332 | 11.1 | 85/729 | 11.7 |
| **Tingling in hands/feet** |  | 12/92 | 13.0 | 61/310 | 19.7 | 68/324 | 21.0 | 141/726 | 19.4 |
| **Feeling swollen** |  | 4/92 | 4.3 | 40/300 | 30.6 | 37/328 | 11.3 | 81/720 | 11.3 |
| **Hot flashes/sweating episodes** |  | 21/93 | 22.6 | 94/307 | 30.6 | 57/332 | 17.2 | 172/732 | 23.5 |
| **Vertigo** |  | 5/94 | 5.3 | 59/306 | 19.3 | 71/328 | 21.6 | 135/728 | 18.5 |
| **Memory/concentration** |  | 13/95 | 13.7 | 64/304 | 21.1 | 68/333 | 20.4 | 145/732 | 19.8 |
| **Sexual problems** |  | 3/94 | 3.2 | 20/305 | 6.6 | 37/320 | 11.6 | 60/719 | 8.3 |
